# Altered HLA Class I Profile Associated with Type A/D Nucleophosmin Mutation Points to Possible Anti-Nucleophosmin Immune Response in Acute Myeloid Leukemia

**DOI:** 10.1371/journal.pone.0127637

**Published:** 2015-05-20

**Authors:** Kateřina Kuželová, Barbora Brodská, Ota Fuchs, Marie Dobrovolná, Petr Soukup, Petr Cetkovský

**Affiliations:** 1 Department of Proteomics, Institute of Hematology and Blood Transfusion, Prague, Czech Republic; 2 Department of Genomics, Institute of Hematology and Blood Transfusion, Prague, Czech Republic; 3 Department of HLA, Institute of Hematology and Blood Transfusion, Prague, Czech Republic; 4 Clinical Department, Institute of Hematology and Blood Transfusion, Prague, Czech Republic; Queen's University Belfast, UNITED KINGDOM

## Abstract

Nucleophosmin 1 (*NPM1*) mutations are frequently found in patients with acute myeloid leukemia (AML) and the newly generated sequences were suggested to induce immune response contributing to the relatively favorable outcome of patients in this AML subset. We hypothesized that if an efficient immune response against mutated nucleophosmin can be induced *in vivo*, the individuals expressing HLA alleles suitable for presenting NPM-derived peptides should be less prone to developing AML associated with *NPM1* mutation. We thus compared HLA class I frequencies in a cohort of patients with mutated *NPM1* (63 patients, NPMc+), a cohort of patients with wild-type *NPM1* (94 patients, NPMwt) and in normal individuals (large datasets available from Allele Frequency Net Database). Several HLA allelic groups were found to be depleted in NPMc+ patients, but not in NPMwt compared to the normal distribution. The decrease was statistically significant for HLA B*07, B*18, and B*40. Furthermore, statistically significant advantage in the overall survival was found for patients with mutated *NPM1* expressing at least one of the depleted allelic groups. The majority of the depleted alleles were predicted to bind potent NPM-derived immunopeptides and, importantly, these peptides were often located in the unmutated part of the protein. Our analysis suggests that individuals expressing specific HLA allelic groups are disposed to develop an efficient anti-AML immune response thanks to aberrant cytoplasmic localization of the mutated NPM protein.

## Introduction

Nucleophosmin 1 (*NPM1*) mutations are the most common single gene abnormality detected in adult acute myeloid leukemia (AML) and might represent the initiating event for AML development in about 60% of AML patients without causative cytogenetic abnormalities [1]. Insertion or duplication of small base stretches results in a shift of the open reading frame and in the generation of unique aminoacid sequence at the C-terminus of the protein [2]. Subsequent loss of affinity to cell nucleolar structures as well as the creation of an additional nuclear export signal contribute to NPM mislocalization to the cell cytoplasm [3,4]. The aberrant cytoplasmic localization helped the discovery of *NPM1* mutations [5] and can be used to diagnose patients bearing these mutations (often denoted as NPM1c+ AML). The majority (75 to 80% of cases) of NPM mutations are of type A with C-terminal sequence 286-DLCLAVEEVSLRK. Type D mutation slightly differs in DNA sequence but the resulting aminoacid chain is identical to that of type A [4].

The high relapse rate in AML is associated with persistent residual disease and immunotherapy represents an attractive candidate tool for disease eradication. Nucleophosmin C-terminal mutations are well-defined molecular alterations which are restricted to AML [6] and generate novel and thus potentially immunogenic epitopes. The positivity for *NPM1* mutation was found to be favorable prognostic factor [7,8] which may be associated with immune response induction. Using *in vitro* assays, several studies analyzed selected peptides from NPMc+ C-terminus as to their binding to the most common HLA alleles or their potential to activate cytotoxic T-lymphocytes [9–12]. However, to our knowledge, no evidence of an efficient *in vivo* immune response to mutated NPM has been reported to date and suitable immunopeptides as well as the appropriate HLA molecules for their presentation still remain to be determined.

In this work, we document existing differences in HLA class I profiles between AML patients with NPMc+ and normal population and correlate them with HLA context of predicted NPM-derived immunopeptides. Our analysis supports the hypothesis that an efficient anti-nucleophosmin immune response is spontaneously elicited *in vivo* and suggests the most important alleles for presentation of NPM-derived peptides.

## Material and Methods

### Patients

A total of 302 samples from AML patients were tested for *NPM1* C-terminal mutations at the Institute of Hematology and Blood Transfusion (Prague, Czech Republic) since the year 2001. Of those, 152 samples were negative (wild-type), mutation type A was found in 114 samples (38% of total, 76% of mutated) and mutation type D in 10 samples. Mutations of other types were found in the remaining 26 samples.

### Ethics Statement

All patients signed written informed consent to the use of their biological material for research purposes in agreement with the Declaration of Helsinki. The study has been approved by the Ethics Committee of the Institute of Hematology and Blood Transfusion of the Czech Republic.

### Standard treatment protocol

Induction therapy 7+3 (AraC, daunorubicin) is followed by 3–4 cycles of consolidation therapy (HIDAC) in responding patients. The induction cycle 7+3 is repeated in case of persisting residual disease or when 5–10% blasts are detected in the bone marrow aspirates on day 14 or 21. Reinduction/salvage therapy (FLA/Ida) is given to patients with refractory disease. Allogeneic stem cell transplantation is indicated in the following cases: disease refractory to 7+3 therapy, achievement of second complete remission after disease relaps, higher-risk disease, Flt3-ITD positivity.

### Detection of NPM1 exon 12 mutations

Mononuclear cells were isolated from the bone marrow or from the peripheral blood of AML patients. Total RNA was prepared with RNA-Bee—RNA isolation reagent (TEL- TEST, Inc.) according to the manufacturer’s instruction. Complementary DNA PCR templates were generated from total RNA (2 μg per reaction) by SuperScript II RNase H reverse transcriptase (Invitrogen). Primers for amplification of a segment of *NPM1* cDNA comprising exons 11 and 12 were the following: forward 5´-GGTGGTTCTCTTCCCAAAGT-3´ and reverse 5´-AACATTTATCAAACAC GGTA-3´[13]. PCR products from the amplification were electrophoresed on 2% agarose gels, electroeluated from the pieces of gel, purified and sequenced using Genome Lab DTCS Quick Start Kit and Beckman Coulter CEQ 3000 DNA sequencer. Gen Bank Accessions NM_002520.6 (Homo sapiens NPM1 transcript variant 1 mRNA) were used for evaluation of obtained sequences.

### HLA typing

Patients with AML indicated for related or unrelated hematopoietic stem cell transplantation are routinely typed in class I loci (HLA-A, -B, -C) and in class II loci (-DRB1, -DQB1) on high-resolution level. We use combination of sequence-based typing (SBT) and polymerase chain reaction with sequence-specific primers (PCR-SSP) to solve ambiguous results and all null alleles are excluded [14–15].

### Immunopeptide prediction

The MHC I binding predictions were made on 6/19/2014 using the IEDB analysis resource Consensus tool [16] which combines predictions from ANN [17–18], SMM [19] and comblib [20]. The cut-off value for IEDB percentile rank was set to 1%. The sequence of type A mutated nucleophosmin was used for all analyses.

### Statistical analysis

The frequencies of the individual class I allelic groups in patients with mutated NPM and with wild-type NPM were compared with normal frequencies of these allelic groups using contingency tables. A p-value of less than 0.05 was considered indicative of a statistically significant difference between groups. Survival curves were created and statistically evaluated using GraphPad Prism software. This software was also used to evaluate the association between changes in HLA-B frequency distribution and the predicted ability of HLA-B alleles to bind NPM-derived immunopeptides (Spearman non-parametric test).

## Results

**Fig 1:**
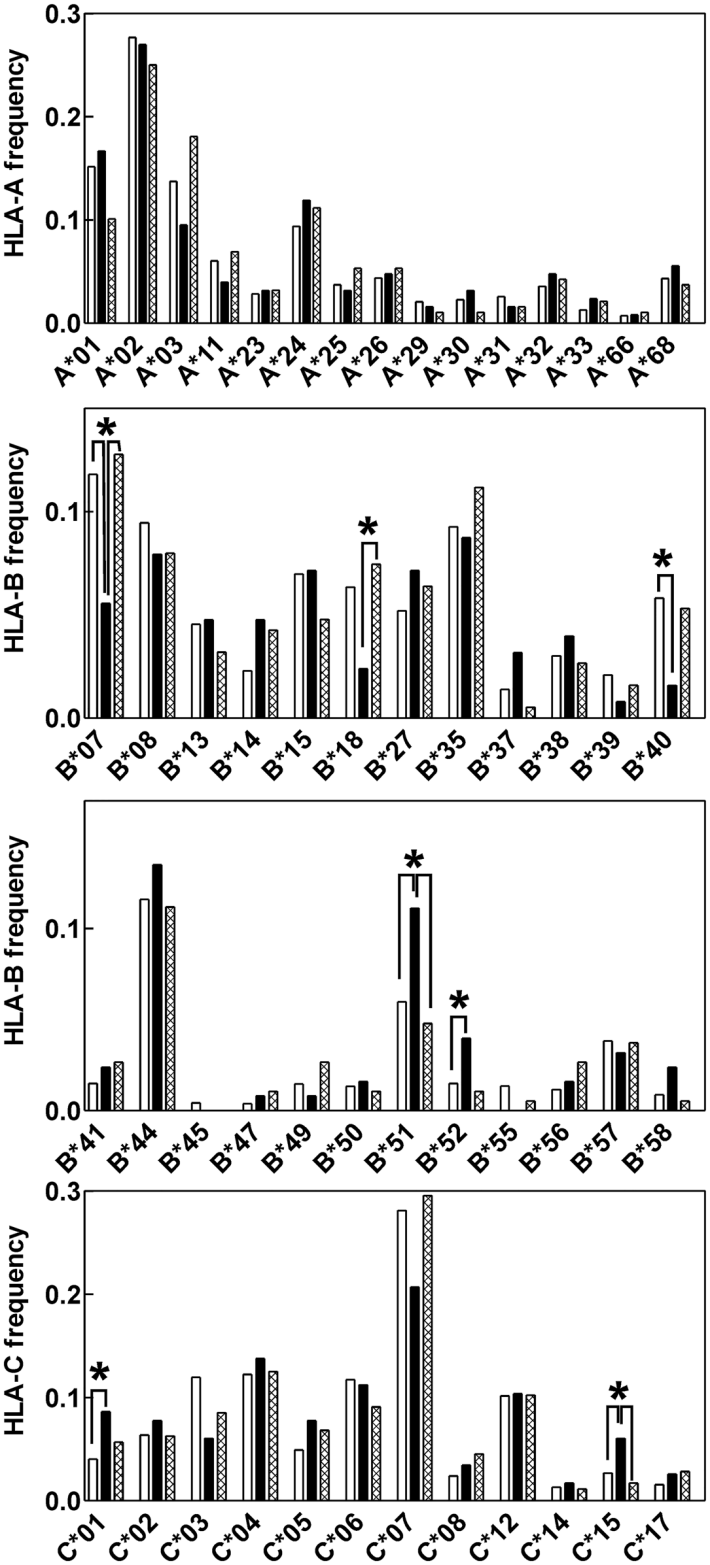
Comparison of HLA class I profiles in AML patients and in normal individuals. HLA class I profiles in normal European population (clear bars), in AML patients with C-terminal *NPM1* mutations (black bars) and in AML patients with wild-type *NPM1* (cross-hatched bars). The normal allele distribution is represented by the means from two large datasets obtained from neighbouring white populations. Differences in allele frequencies between groups were statistically evaluated using contingency tables. Statistically significant differences (p < 0.05) are marked with asterisks.

Results of HLA typing were available for 63 AML patients bearing type A or type D nucleophosmin mutations (group NPMc+) and for 94 AML patients with wild-type nucleophosmin (NPMwt). HLA I allele frequencies for the two AML patient cohorts were calculated and compared with allele distribution in two merged large datasets obtained from neighbouring European populations: 2907 healthy individuals from Poland and 1698 normal samples from Germany-Austria minority, both of them accessible at Allele Frequency Net Database [21]. As shown in Fig 1, the frequencies of some alleles were reduced in NPMc+ but not in NPMwt AML. In particular, statistically significant decrease was found for B*07, B*18, and B*40 allelic groups. Less marked depletion was also observed for A*03, A*11, B*39, C*03 and C*07. On the other hand, statistically significant increase in B*51, B*52, C*01 and C*15 was found in NPMc+ patient group compared to the normal samples. The results of the statistical evaluation for all allelic groups (p values and odds ratios) are given in the Supplementary Material (S1 Table).

We also examined the frequence distribution of HLA class II and noted statistically significant decrease of DRB1*04 in NPMc+ compared to NPMwt patient group (S1 Fig). Interestingly, low HLA-DR4 (the serological equivalent of DRB1*04) frequency was reported previously in chronic myeloid leukemia patients [22].

The depletion of certain HLA class I alleles in NPMc+ AML group indicated that individuals with specific HLA types may develop an efficient anti-NPM immune response and be more resistant to AML development. Subsequently, we searched for possible effect of HLA type on AML patient outcome. In a retrospective analysis, NPMc+ AML patients with complete data (N = 60) were separated into two groups: those bearing at least one of the depleted allelic groups (i.e. A*03, A*11, B*07, B*18, B*39, B*40, C*03 or C*07) and those not having any of these alleles. Group characteristics are specified in Table 1.

**Table 1:** Characteristics of NPMc+ AML patient groups.

	group 1	group 2
Patient number	N = 41	N = 19
Age at diagnosis: mean	50.6	49.2
Age at diagnosis: range	24 to 64	24 to 65
% of Flt3-ITD positive cases	51% (20/39, 2 not tested)	44% (7/16, 3 not tested)
% of DNMT3 mutated cases	71% (25/35, 6 not tested)	75% (12/16, 3 not tested)
% of transplanted	59% (24/41)	26% (5/19)
% of relapsed	44% (18/41)	63% (12/19)

NPMc+ AML patients were divided into two groups on the basis of HLA typing. Group 1 includes patients bearing at least one of the following allelic groups: A*03, A*11, B*07, B*18, B*39, B*40, C*03, C*07. Group 2 includes patients not having any of these alleles. All patients were treated using the same standard protocols.

**Fig 2:**
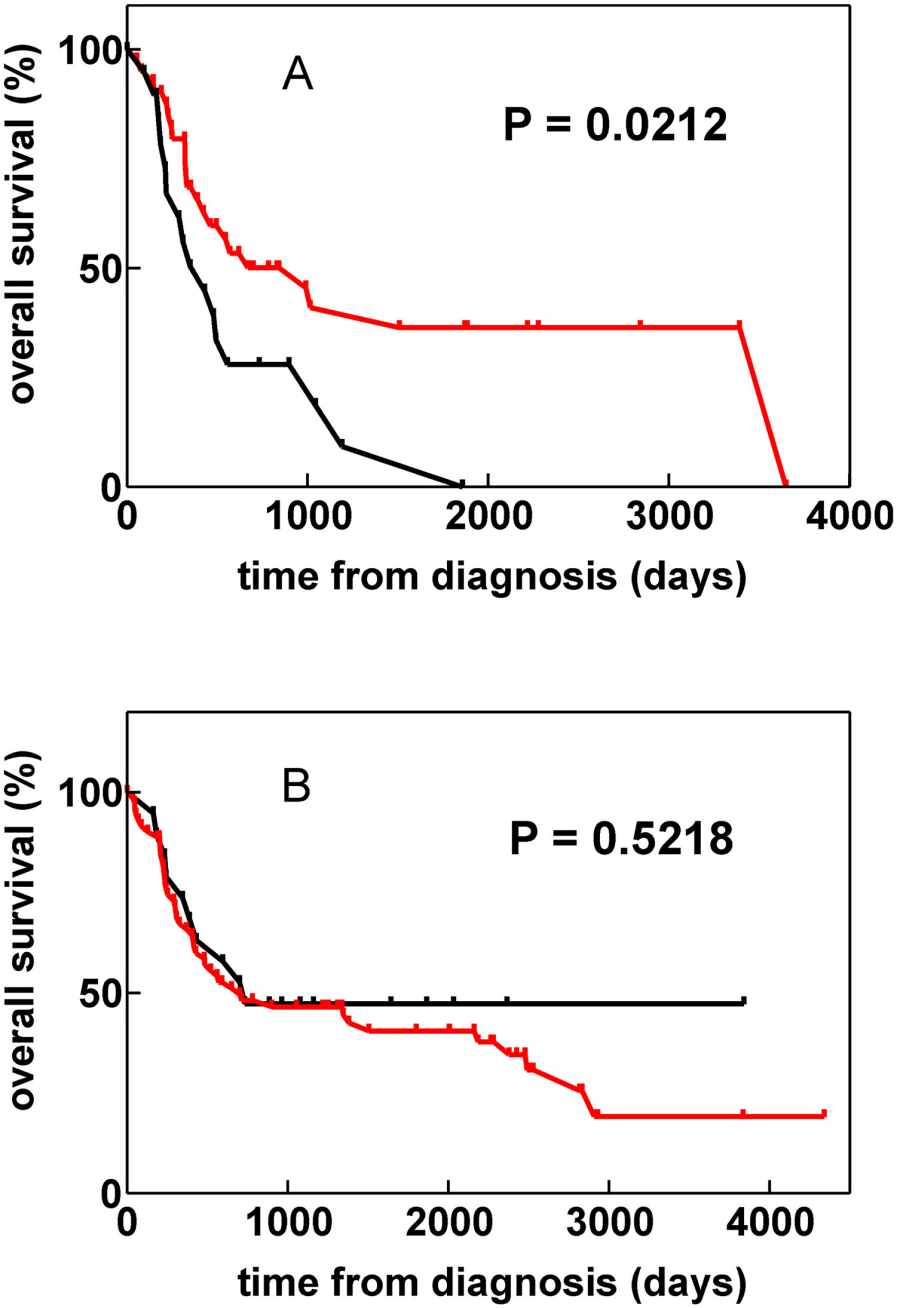
Overall survival of AML patients with different HLA types. Group 1 (red) includes patients bearing at least one of the following allelic groups: HLA-A*03, A*11, B*07, B*18, B*39, B*40, C*03 or C*07. Patients in the group 2 (black) express none of these HLA alleles. The survival time from the day of diagnosis was evaluated. All patients were treated using the same standard protocol. Panel A: Patients with mutated *NPM1* (NPMc+). The basic characteristics of the NPMc+ patient groups are given in Table 1. Panel B: Patients with wild-type *NPM1*.

The analysis of the overall survival revealed significantly better prognosis for patients expressing the selected HLA alleles (Fig 2A). On the contrary, HLA type had no effect on the overall survival in the group of patients with wild-type NPM (Fig 2B).

Immunoepitopes derived from mutated nucleophosmin and restricted to HLA class I molecules were predicted using the Immune Epitope Database (IEDB). We first searched for leukemia-specific peptides involving the mutated C-terminal sequence (beginning at position 288). The cut-off value for percentile rank was set to 1% as recommended by the authors of the site and peptides of 8–11 aminoacids were screened for the probability to be exposed on HLA-A, -B and -C alleles. Table 2 lists all peptides meeting the given criteria. The strongest epitope was predicted to be restricted to C*03 allelic group. Compared to this epitope, peptides binding to B*40 had markedly lower affinity (two orders of magnitude for IC50 value) and poorer score (percentile rank 0.9). In addition, no immunopeptide from the mutated part of the protein was found for B*07 and B*18 alleles.

**Table 2:** Immunopeptides (8-11AA) derived from the mutated C-terminus of NPM.

allele	peptide length	start	end	sequence	perc. rank	IC50 (nM)	SYFPEITHI score
C*03:03	8	289	296	LAVEEVSL	0.3	3	-
C*03:04	8	289	296	LAVEEVSL	0.4	-	-
B*35:03	8	289	296	LAVEEVSL	0.5	2264	-
B*37:01	11	281	291	QEAIQDLCLAV	0.7	-	-
A*11:01	9	290	298	AVEEVSLRK	0.75	35	27
A*68:01	9	289	297	LAVEEVSLR	0.8	30	20
B*40:01	9	281	289	QEAIQDLCL	0.9	313	24
B*49:01	11	281	291	QEAIQDLCLAV	0.9	-	-
B*40:02	11	281	291	QEAIQDLCLAV	0.9	360	-

Immune Epitope Database (IEDB) was used to predict immunopeptides derived from the mutated sequence of type A mutated NPM. The cutoff value of percentile rank was set to 1%. IC50 values were calculated using ANN tool embedded in IEDB. When available, scores according to SYFPEITHI database are also given for the predicted peptides.

The aberrant cytoplasmic localization of the mutated protein may, however, favor NPM processing and presentation of all NPM-derived peptides. We thus performed similar search for high-affinity binding peptides (i.e., IC50 less than 50 nM) from the whole NPM sequence. As it follows from Table 3 and Supplementary material (S2 Fig), an excellent correlation was found for HLA-B alleles in this case. The strongest epitopes are predicted to be formed in the context of B*07, B*18, B*39 and B*40 alleles and a marked decrease in the corresponding frequencies was actually detected for all of them (see Fig 1). On the other hand, while HLA-A and HLA-C-restricted epitopes should exist according to the prediction, no decrease in the frequency of the corresponding alleles was usually noted.

**Table 3:** Predicted immunopeptides (8–11 AA) derived from the unmutated NPM sequence.

allele	peptide length	start	end	sequence	perc. rank	IC50 (nM)	SYFPEITHI score
**HLA-A**							
A*68:01	9	265	273	EAKFINYVK	0.2	8	19
A*30:01	9	221	229	RSKGQESFK	0.2	4	-
A*68:01	10	268	277	FINYVKNCFR	0.3	8	15
A*30:01	8	195	202	SIRDTPAK	0.3	7	-
A*68:01	11	3	13	DSMDMDMSPLR	0.3	10	19
A*68:01	9	93	101	EITPPVVLR	0.35	12	28
A*30:01	9	194	202	KSIRDTPAK	0.4	8	-
A*31:01	10	268	277	FINYVKNCFR	0.4	18	-
A*31:01	9	269	277	INYVKNCFR	0.5	12	-
A*02:01	9	17	25	YLFGCELKA	0.6	12	22
A*11:01	9	194	202	KSIRDTPAK	0.65	27	21
A*30:01	8	248	255	KAKMQASI	0.7	23	-
A*68:01	11	63	73	EAMNYEGSPIK	0.7	26	18
A*68:01	9	81	89	MSVQPTVSL	0.8	15	8
A*68:01	8	17	24	YLFGCELK	0.8	31	-
A*30:01	11	101	111	RLKCGSGPVHI	0.8	35	-
A*11:01	10	64	73	AMNYEGSPIK	0.85	48	19
A*30:01	8	73	80	KVTLATLK	0.9	35	-
A*30:01	8	148	155	GSKVPQKK	0.9	34	-
**HLA-B**							
B*39:01	10	40	49	HQLSLRTVSL	0.1	8	17
B*40:01	9	92	100	FEITPPVVL	0.2	8	30
B*07:02	10	70	79	SPIKVTLATL	0.2	14	22
B*18:01	9	36	44	DENEHQLSL	0.25	13	25
B*07:02	10	219	228	TPRSKGQESF	0.25	29	18
B*39:01	9	92	100	FEITPPVVL	0.3	18	16
B*08:01	9	41	49	QLSLRTVSL	0.3	40	26
B*40:01	11	62	72	AEAMNYEGSPI	0.3	16	-
B*40:01	11	92	102	FEITPPVVLRL	0.3	16	-
B*39:01	11	79	89	LKMSVQPTVSL	0.4	28	-
B*39:01	9	276	284	FRMTDQEAI	0.45	19	16
B*07:02	9	10	18	SPLRPQNYL	0.5	31	23
B*07:02	9	70	78	SPIKVTLAT	0.5	19	21
B*39:01	10	70	79	SPIKVTLATL	0.5	39	13
B*39:01	11	39	49	EHQLSLRTVSL	0.5	46	-
B*07:02	11	13	23	RPQNYLFGCEL	0.55	16	-
B*15:01	8	221	228	RSKGQESF	0.6	47	-
B*58:01	8	24	31	KADKDYHF	0.6	38	-
**HLA-C**							
C*05:01	8	24	31	KADKDYHF	0.2	3	-
C*15:02	10	142	151	RSAPGGGSKV	0.3	42	-
C*05:01	10	278	287	MTDQEAIQDL	0.3	11	-
C*07:01	8	109	116	VHISGQHL	0.3	27	-
C*06:02	9	276	284	FRMTDQEAI	0.35	35	-
C*05:01	11	32	42	KVDNDENEHQL	0.5	45	-
C*07:01	9	276	284	FRMTDQEAI	0.55	32	-
C*12:03	8	65	72	MNYEGSPI	0.6	23	-
C*12:03	10	63	72	EAMNYEGSPI	0.8	29	-
C*15:02	10	74	83	VTLATLKMSV	0.8	93	-
C*14:02	11	66	76	NYEGSPIKVTL	0.8	26	-
C*03:03	11	47	57	VSLGAGAKDEL	0.8	11	-
C*03:03	9	92	100	FEITPPVVL	0.9	8	-
C*03:03	11	92	102	FEITPPVVLRL	0.9	13	-

Immune Epitope Database (IEDB) was used to predict immunopeptides derived from the whole sequence of type A mutated NPM. The cutoff value of percentile rank was set to 1% and the resulting peptide sets were restricted to high-affinity peptides (IC50 less than 50 nM). When available, scores according to SYFPEITHI database are also given for the predicted peptides.

## Discussion

Mutations occurring at the C-terminus of nucleophosmin create unique aminoacid sequences which might be immunogenic. It has been reported that peptides derived from the mutated part of NPMc+ are able to induce specific T-cell response in *ex vivo* experiments [11]. However, in these experiments, the priming cells were loaded with high amount of synthetic peptide and it is unclear whether the conclusions are applicable to *in vivo* conditions which are more complex and can involve a variety of immunosuppresive mechanisms. We hypothesized that if an efficient immune response against mutated nucleophosmin can be induced *in vivo*, the individuals expressing HLA alleles suitable for presenting NPM-derived peptides should be less prone to developing AML due to NPM1 mutation. Indeed, a few HLA class I alleles had strikingly reduced incidence in NPMc+ patient group compared to healthy controls as well as to the patients with NPMwt (Fig 1).

Differences in HLA class I profiles associated with AML have already been reported in several previous studies which, however, did not focus on patients with NPM mutations. Therefore, only the most prominent changes could have been distinguished. In particular, statistically significant decrease in B18 antigen frequency [23] as well as in B*40 gene frequency [24] have already been detected. Studies from AML patient cohorts regardless of NPM mutation also showed an increase in B49 antigen frequency [25] and a decrease of B*15 gene frequency [24]. We have observed similar trend (not significant) in patients with wtNPM but not in the group with NPMc+ (Fig 1). On the other hand, Bortin et al. reported increased expression of Cw3 and Cw4 antigens in association with AML [26]. This finding has not been confirmed in our analysis by molecular methods. Interestingly, some alleles were found to be over-represented in our NPMc+ patient cohort (B*37, B*51, B*52, C*01, and C*15). These differences were statistically significant for the most of them and indicate that a few HLA types may be predisposed to NPMc+ AML.

As the marked depletion of several allelic groups from HLA distribution profile of NPMc+ AML patients suggests that anti-NPM immune response really occurs *in vivo*, we searched for possible effects of HLA type on patient outcome in this group. Indeed, the analysis of the overall survival showed that patients bearing at least one of the depleted alleles have better prognosis in comparison with those who do not have such predisposition (Fig 2A). As expected, no such association was found in the group of AML patients without mutation in *NPM1* (Fig 2B).

Although the basic characteristics of the groups 1 and 2 shown in Fig 2A were similar (Table 1), we noted a difference in the fraction of transplanted patients. As this difference might be associated with the observed difference in the overall survival, we analyzed in detail the reasons for unusually low number of transplantations (5/19) in the group 2: although additional 7 patients were indicated due to Flt3-ITD positivity or relaps (12/19 in total), the transplantation could not be performed mainly due to unfavorable leukemia status (6/7, 1 patient had atypical pneumonia and flu). Also, hyperleukocytosis was often present in the patients belonging to the group 2. In general, a large part of group 2 is formed by patients with severe disease course. Hypothetically, this fact might be related to the absence of T-cell-mediated immune response due to the unsuitable HLA class I profile.

We subsequently used a prediction tool to check for the existence of peptides which would be derived from the mutated NPM part and which could be presented in the context of these particular alleles. Surprisingly, only the strongest predicted immunoepitopes (an octamer binding to C*03 and a nonamer binding to A*11, see Table 2) were found to match with the observed changes in HLA profiles. On the other hand, as it follows from Table 3, the observed decrease in the frequencies of B*07, B*18, B*39 and B*40 alleles is likely associated with generation of immunoepitopes containing peptides from unmutated NPM sequences. This implies that the immunogenicity of NPMc+ is not only due to novel peptide sequences but also to the cytoplasmic localization of the mutated protein which presumably assures more efficient processing by the proteasome.

We observed good correlation between reduction of particular HLA alleles in NPMc+ AML and peptides with the best score (less or equal to 0.3) and with high binding affinity (IC50 less than 50 nM). Significant association of changes in HLA frequency distribution due to NPM1 mutation with predicted immunoepitopes was found for HLA-B alleles (S2 Fig). While strong immunoepitopes were predicted to be exposed on HLA-A*30 and 68, no decrease of their frequency was observed in NPMc+ patients. However, these alleles have low incidence in general and this result could thus be false negative. The prediction did not give satisfactory results for HLA-C alleles. While the presumed strongest immunoepitopes are formed in the context of C*05, a decrease occurred in C*03 and C*07 frequency. This may be due to low immunogenicity of some peptides which is not included in the prediction score. Also, as the epitopes seem to be often derived from the unmutated part of nucleophosmin, specific cytotoxic T-lymphocytes against some strong NPM-derived immunoepitopes might be formed prior to the emergence of NPM C-terminal mutation and inhibited by natural mechanisms of preventing immune response against self-antigens. We have also noted that some peptides were predicted to bind to more than one HLA allele—e.g. the nonamer FEITPPVVL (aminoacids 92 to 100) to B*39, B*40 and C*03 and the decamer SPIKVTLATL (aminoacids 70 to 79) to B*07 and B*39.

Greiner et al. have recently found significant differences in survival of AML patients between small groups (25 patients in total) with different *in vitro* responses to stimulation with two peptides derived from mutated C-terminal NPM sequences [12]. The response was evaluated on the basis of cytokine production after incubation of peptide-stimulated CD8+ T-lymphocytes with target cells (T2 cell line) loaded with the peptide. However, high response rate had been found previously in healthy volunteers, too, using the same assay and the same peptides [11]. We thus believe that the *in vitro* assay used in these studies rather reflects the general ability of patient T-cells to be stimulated by a foreign peptide. This may actually be important for the development of specific T-cell response which would explain the observed difference in the outcome of responding vs non-responding patients. However, any other NPM-derived peptide may have been responsible for the induction of *in vivo* anti-NPM immune reaction in responding patients.

The search for immunopeptides suitable for immunotherapy often focuses on ligands of the most frequent allelic group, HLA-A*02. The peptide AIQDLCLAV (derived from the mutated C-terminus) that had been tested in the above cited *ex vivo* experiments [11] has a predicted IC50 value of 97 nM for binding to A*02 allele and a poor overall score (IEDB percentile rank of 2.3%). We have found one nonamer derived from the unmutated part of NPM which is predicted to have high affinity to A*02 (IC50 of 12 nM) but its percentile score (0.6) ranks it to less efficient peptides. Accordingly, no change at A*02 was observed in our HLA profiles (Fig 1) and no evidence of humoral immune response against HLA-A2-binding peptides was found *in vivo* [9]. Thus, HLA-A*02 does not seem to be the proper context for presenting NPM-derived peptides. However, using the normal frequencies and the calculus of probabilities, it can be estimated that 87% of white population express at least one of the alleles which are depleted in NPMc+ patients and can thus be supposed to be suitable for presenting NPM-derived peptides (A*03, A*11, B*07, B*18, B*39, B*40, C*03 or C*07).

In conclusion, although our evidences are indirect, they strongly suggest that *NPM1* mutations are associated with the induction of efficient spontaneous immune response *in vivo*. This response may rescue a large part of individuals expressing appropriate HLA alleles from developing AML initiated by nucleophosmin mutations and helps achieve durable response to treatment in the remaining cases. The immunogenicity of the mutated protein is probably due to its aberrant cytoplasmic localization and involves mainly non-mutated aminoacid sequences. Although our results need to be validated on larger patient cohorts, they indicate that patients bearing suitable alleles may benefit from immunomodulatory treatment in combination with standard chemotherapy. As our analysis provided useful hints as to suitable NPM-derived immunopeptides, our future project will include attempts to detect specific cytotoxic T-lymphocytes recognizing these peptides in AML patients.

## Supporting Information

S1 Fig Comparison of HLA class II profiles in AML patients with mutated and wild-type NPM1. (PDF)

S2 Fig Statistical evaluation of the association between decreased HLA-B frequences and predicted NPM-derived immunoepitopes. (PDF)

S1 Table Results of statistical evaluation of differences in HLA class I profile. (PDF)
