# Supplementary material for: Altered HLA Class I Profile Associated with Type A/D Nucleophosmin Mutation Points to Possible Anti-Nucleophosmin Immune Response in Acute Myeloid Leukemia
Source: PLoS One. 2015 May 20;10(5):e0127637. doi: 10.1371/journal.pone.0127637 (PMC4439052; doi:10.1371/journal.pone.0127637)

Figure S1: **Comparison of HLA class II profiles in AML patients with mutated and wild-type NPM1**. HLA class II allele distribution in AML patients with C-terminal *NPM1* mutations (black bars) and in AML patients with wild-type *NPM1* (cross-hatched bars). Differences in allele frequencies between groups were statistically evaluated using contingency tables. The only statistically significant difference ($p < 0.05$) is marked with the asterisk.

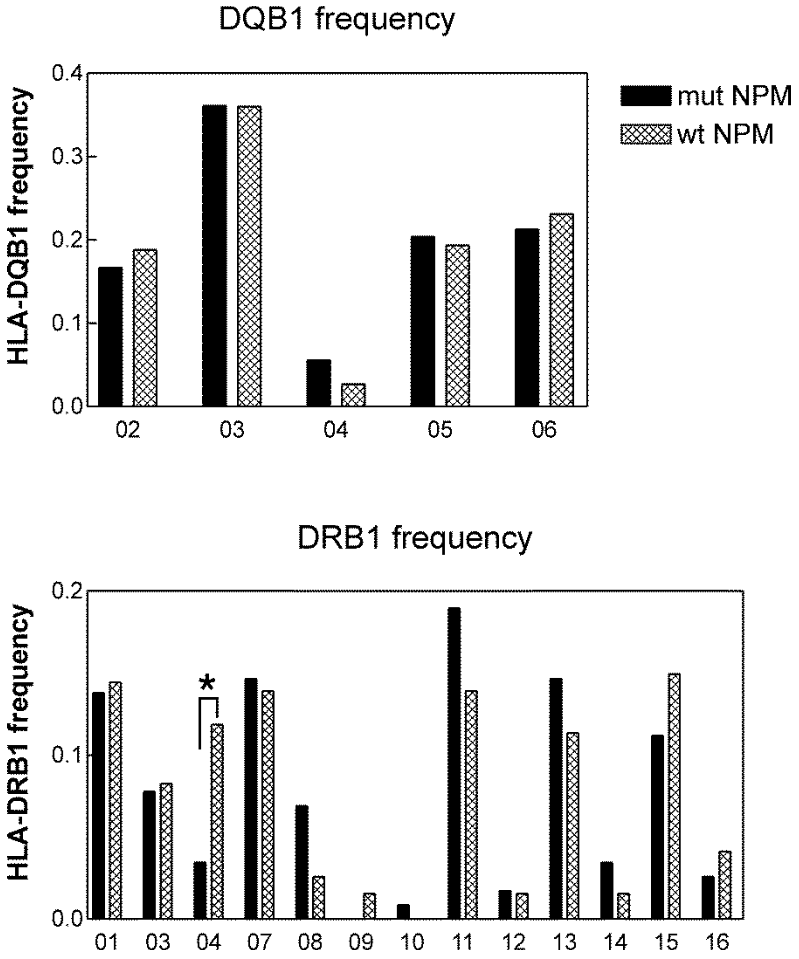

Supplement: S1 Fig — (PDF) [file pone.0127637.s001.pdf]
