# Supplementary material for: Altered HLA Class I Profile Associated with Type A/D Nucleophosmin Mutation Points to Possible Anti-Nucleophosmin Immune Response in Acute Myeloid Leukemia
Source: PLoS One. 2015 May 20;10(5):e0127637. doi: 10.1371/journal.pone.0127637 (PMC4439052; doi:10.1371/journal.pone.0127637)
Supplement: S2 Fig — (PDF) [file pone.0127637.s002.pdf]

Figure S2: Statistical evaluation of the association between decreased HLA-B frequences and predicted NPM-derived immunoepitopes.

Using Spearman non-parametric test, we compared the change in HLA-B frequency (ratio of frequency in NPMc+ patient cohort to normal frequency) with the lowest percentile rank (according to IEDB) among all high-affinity immunopeptides (IC50 less than 50 nM) binding to the corresponding allele. Alleles with frequency higher than 0.02 in normal population were included in the analysis. Percentile rank 1.0 (the cut-off value used for the prediction) was attributed to alleles which were predicted not to have high-affinity ligands.

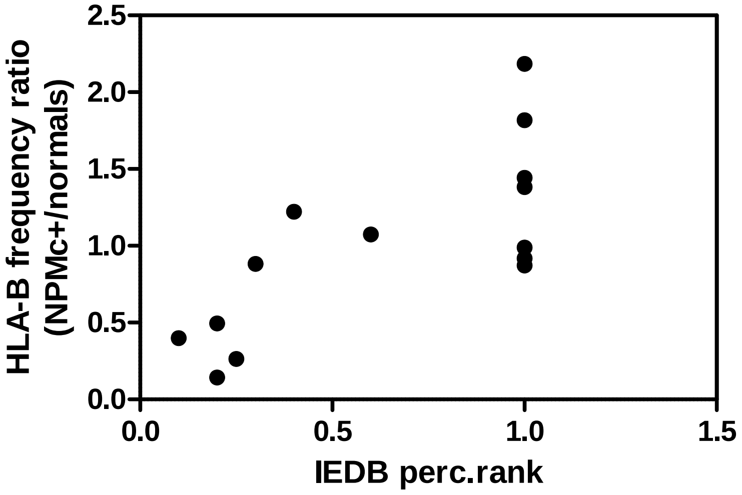

p = 0.0021
correl. coef. = 0.7473
