# Supplementary material for: Altered HLA Class I Profile Associated with Type A/D Nucleophosmin Mutation Points to Possible Anti-Nucleophosmin Immune Response in Acute Myeloid Leukemia
Source: PLoS One. 2015 May 20;10(5):e0127637. doi: 10.1371/journal.pone.0127637 (PMC4439052; doi:10.1371/journal.pone.0127637)
Supplement: S1 Table — (PDF) [file pone.0127637.s003.pdf]

Table S1: Statistical evaluation of the observed differences between HLA class I allelic group frequency in AML patients and controls (the results are shown in Figure 1). The frequency of the individual allelic groups in patients with mutated (mutNPM) and wild-type (wtNPM) nucleophosmin were compared with that of healthy individuals using contigency tables.

	mutNPM vs control		wtNPM vs control	
	p value	odds ratio (OR)	p value	odds ratio (OR)
A*01	0.6405	1.12	0.0579	0.63
A*02	0.8704	1.03	0.4224	0.87
A*03	0.2411	0.70	0.0914	1.39
A*11	0.3973	0.68	0.6174	1.16
A*23	0.7412	1.19	0.7676	1.13
A*24	0.2536	1.38	0.4181	1.21
A*25	0.8231	0.89	0.2637	1.45
A*26	0.7420	1.15	0.5381	1.23
A*29	0.7565	0.80	0.3366	0.51
A*30	0.4466	1.48	0.2707	0.46
A*31	0.5339	0.64	0.4035	0.61
A*32	0.4100	1.42	0.6248	1.2
A*33	0.2450	1.99	0.3156	1.68
A*66	0.8906	1.15	0.5963	1.47
A*68	0.4257	1.37	0.6910	0.86
B*07	**0.0312**	**0.44**	0.6887	1.09
B*08	0.5666	0.83	0.4990	0.83
B*13	0.9106	1.05	0.3793	0.69
B*14	0.0740	2.12	0.0861	1.89
B*15	0.9420	1.03	0.2467	0.67
B*18	0.0695	0.36	0.5482	1.19
B*27	0.3361	1.40	0.4774	1.24
B*35	0.8354	0.94	0.3833	1.23
B*37	0.1013	2.31	0.3154	0.38
B*38	0.5446	1.32	0.7744	0.88
B*39	0.3094	0.37	0.6357	0.76
B*40	**0.0441**	**0.26**	0.7824	0.91
B*41	0.4272	1.60	0.2087	1.79
B*44	0.5146	1.19	0.8572	0.96
B*45	0.4634	0 - 9.5	0.3704	0 - 6.35
B*47	0.4566	2.12	0.1466	2.84
B*49	0.5412	0.54	0.183	1.86
B*50	0.8004	1.20	0.7585	0.8
B*51	**0.0172**	**1.98**	0.5084	0.79
B*52	**0.0285**	**2.71**	0.6284	0.71
B*55	0.1892	0 - 2.85	0.3356	0.39
B*56	0.6522	1.38	0.0651	2.35
B*57	0.7071	0.82	0.9434	0.97
B*58	0.0814	2.76	0.6185	0.61
C*01	**0.0145**	**2.25**	0.2777	1.44
C*02	0.5488	1.24	0.9470	0.98
C*03	0.0514	0.47	0.1679	0.69

C*04	0.6118	1.15	0.9132	1.03
C*05	0.1630	1.63	0.2523	1.42
C*06	0.5541	1.26	0.2858	0.75
C*07	0.0915	0.62	0.6819	1.07
C*08	0.4697	1.45	0.0738	1.94
C*12	0.9454	1.02	0.9735	1.01
C*14	0.6923	1.33	0.8505	0.87
C*15	0.0297	**2.34**	0.4313	0.63
C*17	0.3927	1.66	0.1941	1.83
